# Assessment of the microbiome during bacteriophage therapy in combination with systemic antibiotics to treat a case of staphylococcal device infection

**DOI:** 10.1186/s40168-021-01026-9

**Published:** 2021-04-14

**Authors:** Andre Mu, Daniel McDonald, Alan K. Jarmusch, Cameron Martino, Caitriona Brennan, Mackenzie Bryant, Gregory C. Humphrey, Julia Toronczak, Tara Schwartz, Dominic Nguyen, Gail Ackermann, Anthony D’Onofrio, Steffanie A. Strathdee, Robert T. Schooley, Pieter C. Dorrestein, Rob Knight, Saima Aslam

**Affiliations:** 1 Doherty Applied Microbial Genomics, Department of Microbiology and Immunology at the Peter Doherty Institute for Infection and Immunity, University of Melbourne, Melbourne, Australia; 2 Microbiological Diagnostic Unit Public Health Laboratory, Department of Microbiology and Immunology at the Peter Doherty Institute for Infection and Immunity, University of Melbourne, Melbourne, Australia; 3 Department of Pediatrics, University of California San Diego, La Jolla, CA USA; 4 Skaggs School of Pharmacy and Pharmaceutical Sciences, University of California San Diego, La Jolla, USA; 5 Collaborative Mass Spectrometry Innovation Center, Skaggs School of Pharmacy and Pharmaceutical Sciences, University of California San Diego, La Jolla, USA; 6 Bioinformatics and Systems Biology Program, University of San Diego, La Jolla, USA; 7 Center for Microbiome Innovation, University of California San Diego, La Jolla, CA USA; 8 Antimicrobial Discovery Center, Department of Biology, Northeastern University, Boston, USA; 9 Division of Infectious Diseases and Global Public Health, Department of Medicine, University of California San Diego, La Jolla, USA; 10 Department of Bioengineering, University of California San Diego, La Jolla, CA USA; 11 Department of Computer Sciences and Engineering, University of California San Diego, La Jolla, CA USA

**Keywords:** Bacteriophages, Phage therapy, Microbiome, Metabolomics, *Staphylococcus aureus*

## Abstract

**Background:**

Infectious bacterial diseases exhibiting increasing resistance to antibiotics are a serious global health issue. Bacteriophage therapy is an anti-microbial alternative to treat patients with serious bacterial infections. However, the impacts to the host microbiome in response to clinical use of phage therapy are not well understood.

**Results:**

Our paper demonstrates a largely unchanged microbiota profile during 4 weeks of phage therapy when added to systemic antibiotics in a single patient with *Staphylococcus aureus* device infection. Metabolomic analyses suggest potential indirect cascading ecological impacts to the host (skin) microbiome. We did not detect genomes of the three phages used to treat the patient in metagenomic samples taken from saliva, stool, and skin; however, phages were detected using endpoint-PCR in patient serum.

**Conclusion:**

Results from our proof-of-principal study supports the use of bacteriophages as a microbiome-sparing approach to treat bacterial infections.

Video abstract

**Supplementary Information:**

The online version contains supplementary material available at 10.1186/s40168-021-01026-9.

## Background

*Staphylococcus aureus* is a common commensal of the skin and anterior nares which can cause an array of serious human diseases, ranging from mild skin infection to life-threatening endocarditis and septicemia. The ability of *S. aureus* to rapidly adapt to selective pressures, such as antibiotics, is exacerbated by biofilm formation on implanted medical devices [1]. With increasing incidence of antimicrobial resistance (AMR) and few new antibiotics in the pipeline, there is a growing need to consider non-antibiotic alternatives to treat serious bacterial infections [2]. One such alternative is the use of bacteriophage therapy (BT) [3, 4]. Bacteriophages are viruses that selectively infect and, in the case of lytic phages, kill their target bacterial host. Some have anti-biofilm activity as well which may be helpful for treating device infections. Additional potential advantages include synergy of phage-antibiotic combinations to either directly lyse bacterial host cells, or apply selective pressure that attenuate virulence (e.g., biofilm formation), and/or re-sensitize bacteria to specific antibiotics [5].

We previously reported the case of a 65-year-old male with left ventricular assist device (LVAD) implantation in 2014 for non-ischemic cardiomyopathy. He developed a persistent *Staphylococcus aureus* LVAD infection in 2015 associated with sternal osteomyelitis and recurrent bacteremia despite multiple surgical debridements and prolonged courses of intravenous (IV) antibiotics. The infection persisted and precluded heart transplant surgery, and so the patient was treated with BT as an adjunct to antibiotics initiated in April 2018 [6]. A combination of three anti-staphylococcal bacteriophages (AB-SA01; NCT03395769; Armata Pharmaceuticals) at a dose of 3 × 10^9^ plaque forming units was administered intravenously (IV) as an outpatient every 12 h for 28 days. The patient received concomitant IV cefazolin 2 g every 8 h and oral minocycline 100 mg twice daily [6]. Of note, the patient had been on IV cefazolin for the past 2.5 months prior to the initiation of BT; he also received multiple courses of prolonged IV antibiotics over the past approximately 2.5 years prior to BT. The patient was successfully treated when BT was combined with antibiotics, had negative sternal wound bacterial culture at end of therapy (day 28), and underwent successful heart transplantation a week after completion of BT [6]. In this proof-of-principle study, we aimed to understand possible cascading ecological effects to the patient-microbiome as potential effects of BT on the host-microbiome during phage therapy are not well characterized.

## Results

Patient samples were self-collected throughout the duration of BT, which represented gut, saliva, and skin (nares, axillary, and forehead) microbiomes for analysis; samples were collected every 12 h with the exception of fecal samples, which were once daily. The first set of patient samples was collected within 24 h of commencing BT, and extended to 7 days post-phage therapy (i.e., the day before heart transplant surgery). Amplicon 16S rRNA gene sequencing generated 29,633 reads per sample on average post-quality control processes, while paired-end metagenome sequencing yielded on average 1,547,826 reads at 150 bp per sample at 45% GC content, and with an average Phred score of 38. Weekly serum samples were also collected prior to phage administration, and 15, 30, and 60 min following a dose for qualitative PCR of the phages. End-point PCR of bacteriophage DNA concentrations in patient serum indicated the presence of the three AB-SA01 phages throughout treatment; concentrations peaked on day 29 pre-dose collection for each phages: J-Sa36 at 71.2 ng/PCR reaction, Sa83 at 166 ng/PCR reaction, and Sa87 at 10.8 ng/PCR reaction (Supplementary Table 5).

In an effort to contextualize microbiome data from a single patient, comparative analyses were computed using reference cohorts from the American Gut Project (AGP) [7]. Patient microbiome samples were analyzed in the context of the following cohorts: (i) healthy participants from the AGP with no recent antibiotics, (ii) AGP participants with antibiotic exposure in the past week, and (iii) intensive care unit (ICU) participants [8]. The rationale for including healthy AGP and ICU participants was to determine the spectrum of severity of dysbiosis in the patient’s microbiome.

**Fig. 1:**
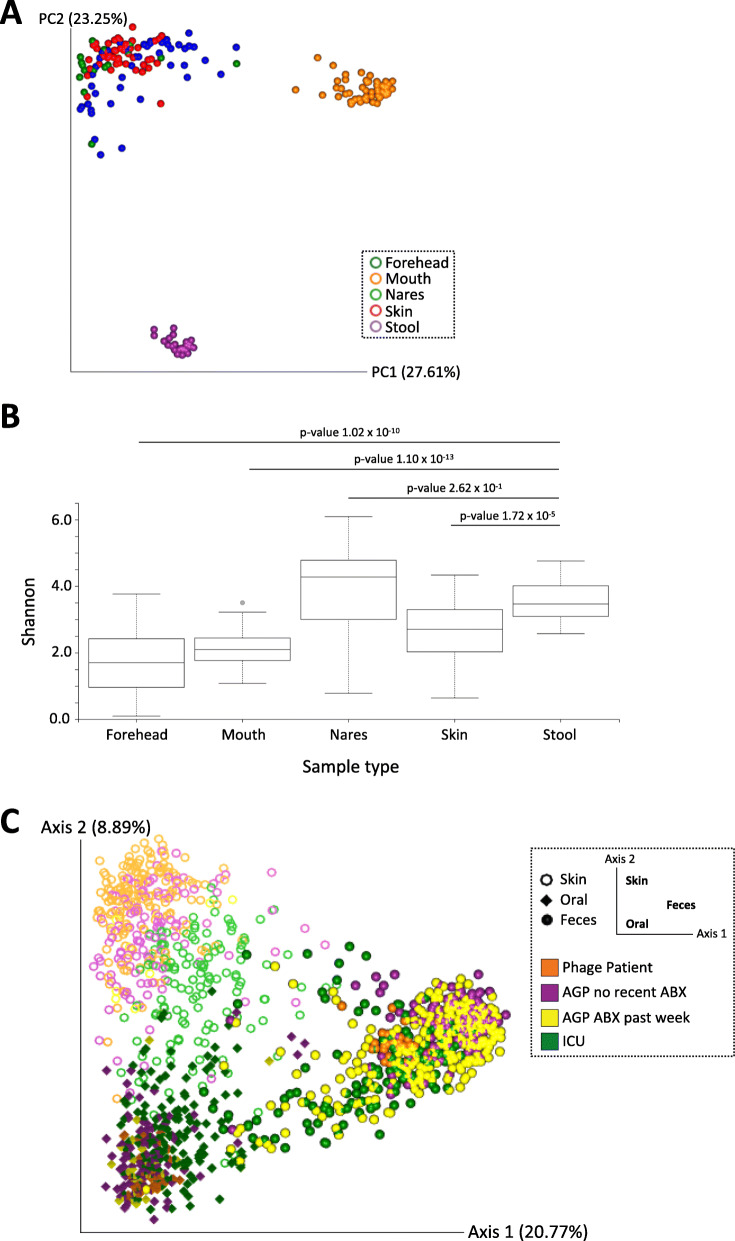
Microbiota analysis of a patient undergoing adjunctive phage therapy, and analysis of patient samples with respect to intensive care unit (ICU) patients, a subset of the American Gut Project (AGP) population that have taken antibiotics within the past week, and a subset of the AGP population who have not taken antibiotics in the past year. **a** Patient microbiota profile. A principal coordinate plot of unweighted UniFrac distances of skin (forehead, blue; nares, green; axilla, red;), oral (mouth, orange), and fecal (stool, purple) samples from phage patient. **b** Comparison of alpha diversity across sample type. Shannon’s diversity for skin (forehead, nares, axilla), oral (mouth), and fecal (stool) samples from phage patient. Boxplots are showing the quartiles (25^th^ and 75^th^ percentiles) on the box, and ± 1.5 interquartile ranges for the whiskers. *P*-values of pairwise Kruskal-Wallis testing are shown for fecal samples compared to other sample types on the figure. A complete list of *p*-values for pairwise comparisons of each sample type is provided in supplementary file. **c** Comparative microbiota analyses. A principal coordinate plot of unweighted UniFrac distances of phage patient (orange) skin (ring), oral (diamond), and fecal (sphere) samples in the context of ICU (green) and AGP (purple or yellow) samples. The sample type is denoted by shape, while sample cohort is denoted by the different colors, within the plot

First-order analyses revealed a clear separation of patient samples according to body site as measured by unweighted UniFrac distances [9] (Fig. 1a). The distinct grouping of patient microbiota within respective body sites is also supported by significant pairwise differences in community richness (Shannon’s index) between sample types; however, there were no significant differences in community richness between nares and stool samples (Fig. 1b and Supplementary Table 2). Samples representing gut and saliva microbiomes demonstrated low level variance in Shannon’s index score across the study, suggesting minimal collateral damage to community richness in response to BT. Comparative microbiota analyses revealed phage-patient samples to be distinct from both ICU and AGP microbiomes; however, they tended to group with ICU samples (albeit not as extreme; Fig. 1c).

**Fig. 2:**
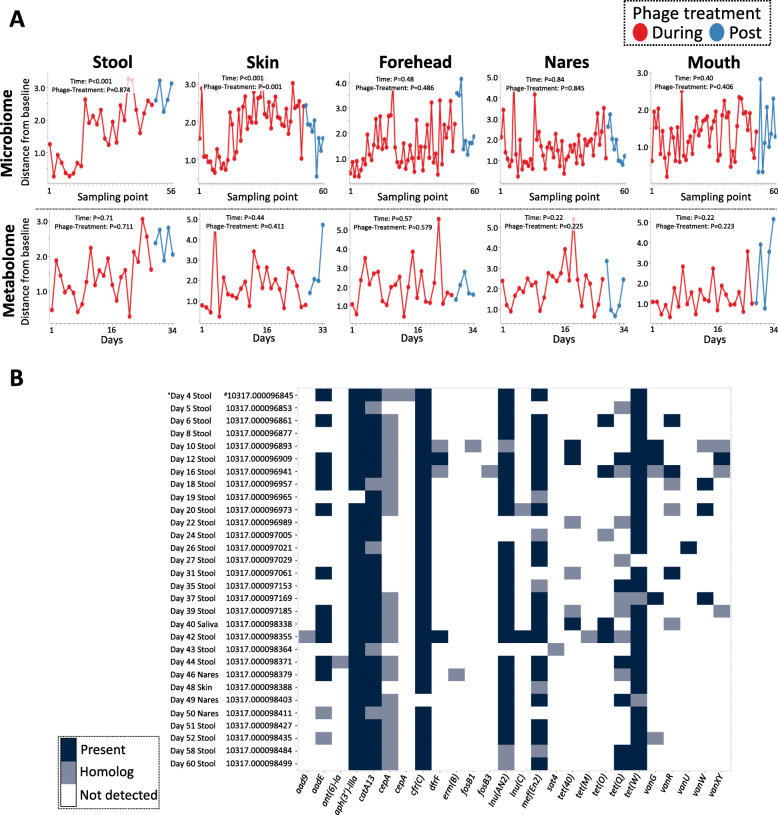
Pairwise distance plots from baseline (pre-treatment) for skin (axilla) microbiome and metabolome samples. **a** Pairwise distances from baseline pre-treatment sample as calculated by a mixed-effects model. The only sample type with significance during phage-treatment is the skin (axilla) microbiome (*P*<0.001). Significance was evaluated by a linear mixed effect model and error bars represent standard error from the mean. **b** Heatmap. Presence of antimicrobial resistance genes and/or its homologs detected in metagenomic samples. Present is defined as 90% gene coverage and 90% nucleotide identity; homolog is defined as 70% gene coverage and 70% nucleotide identity. ^^^Sample time point, and sample type. ^#^Qiita study ID followed by Qiita sample ID. This ID can be used to search https://qiita.ucsd.edu/ for the primary derived data and associated metadata

Longitudinal analyses, including pairwise distance calculations from baseline (pre-treatment) and mixed effects statistical testing, of patient microbiome and metabolomes showed significant changes in axilla skin samples over time (Fig. 2a); the remaining sample types were relatively unchanged over time (Fig. 2a). Specifically, log-ratio calculations of the sub-operational taxonomic unit (sOTU) classified as *Staphylococcus*, and the highly proliferative skin commensal *Corynebacterium* demonstrated significant changes over time (*P* < 0.001), and within the phage treatment phase (*P*< 0.001) (Supplementary Figure 3A). Metabolites with opposing loadings (*refer to* Supplementary data for biplot data) revealed significant (*P* = 0.03) temporal and phage treatment responses (Supplementary Figure 3B). Analysis between the longitudinal rolling mean (window size of 6) of log-ratios of metabolite and microbes identified a temporal separation in profile (Supplementary Figure 4). Additionally, a list of key metabolites and their corresponding annotations based on spectral library matching in Global Natural Products Social Molecular Networking (GNPS) are provided in supplementary material. The majority of the metabolites remain unidentified; however, the measured m/z, retention time, and data are available. Genes detected in patient shotgun metagenomic data from fecal (*n* = 25), and representative skin (*n* = 4) and saliva (*n* = 1) samples, include those predicted to encode resistance to the following classes of antibiotics: aminoglycosides, chloramphenicol, extended spectrum beta-lactams, lincosamides, trimethoprim, macrolide, fosfomycin, and vancomycin (*vanG*). Of note is the presence of *tet* genes encoding resistance to tetracyclines (e.g., minocycline) in patient fecal samples.

## Discussion

The novelty and rarity of extensive microbiome assessments of BT patients underpins the importance of our current work towards informing wide-spread clinical use of bacteriophages for treating multidrug resistant bacterial infections. Our main finding was that the case patient’s gut and saliva microbiomes did not change significantly over time when BT was added to pre-existing systemic antibiotics. However, we noted a significant decrease in staphylococci from axillary skin specimens during the course of BT.

Our comparative microbiome analyses take advantage of the dynamic American Gut Project (AGP) data set, which sampled 230 AGP samples at random to represent the core “healthy” sample set, and 115 intensive care unit (ICU) patients totaling 230 samples [7]. The baseline microbiome profile of the case patient at the start of BT was perturbed when compared to control AGP dataset but not as perturbed as the ICU data (Fig. 1c). This observation may be related to the fact that the patient had already been on systemic cefazolin for 2.5 months prior to BT and had been on prolonged courses of systemic antibiotics for more than a year. The severity of impact to the patient microbiome in response to prolonged antibiotic exposure would have profound effects on microbial community composition relative to individuals whom have not taken antibiotics in the past year (AGP control). However, BT was self-administered as an outpatient; patient was not critically ill and received adequate enteral nutrition—this may explain why his microbiome profile was not as “extremely” perturbed as those patients in the ICU (Fig. 1c). We acknowledge that the AGP control samples may not be an optimal comparison for our case patient; however, the comparison allows for semi-quantitative assessment of such a rare dataset. While our results suggest that IV phage therapy directed at *S. aureus* does not impact the gut microbiota in a single patient (Fig. 1), more research is required to better understand the true extent (e.g., leaky gut and translocation of bacterial metabolites through the blood circulatory system) of downstream collateral damage. For example, Hsu et al. [10] demonstrated in a mouse model that phage predation on target bacterial pathogens has cascading effects on the remaining microbial community members and consequences on the gut metabolome. Our metabolomic analyses suggest minimal temporal shift in gut metabolome throughout the course of adjunct phage therapy (Supplementary data); however, downstream ecological effects—particularly, metabolic “hand-off” interactions—including impacts to host immune responses remain to be determined [11].

We noted a significant decrease in staphylococci from axillary skin specimens during the course of BT, which we ascribe to the *S. aureus*-specific phages used for BT. Of note, we detected cefazolin in patient peripheral skin sites; as the patient had been on cefazolin for 2.5 months prior to BT initiation, we do not think that temporal change of staphylococci at this site is related to cefazolin use (Supplementary file). Figure 2a alludes to the selective specificity of AB-SA01 phages for staphylococci as the log-ratios between staphylococci and the highly proliferative skin commensal, *Corynebacterium*, changes significantly in time and during the phage-treatment phase; specifically, a decrease in *Staphylococcus* relative to *Corynebacterium* was observed to be the key driver of microbial shifts (Supplementary Figure 3A). Similarly, we observed significant changes in key metabolites over time (Supplementary Figure 3B) that correlate significantly with staphylococci (Supplementary Figure 4). However, the molecular mechanisms driving the changing metabolite profile remain to be determined and warrant further investigation as phage therapy is more widely utilized; for example, the majority of metabolite features were unannotated, and given these are axilla samples, the changes could reflect the use of different personal care products. Further research is needed as part of clinical trials to better understand the effects of phage therapy.

The need for non-antibiotic alternatives in treating infectious diseases is compounded by the detection of AMR genes in the patient’s gut microbiome. Of note is the presence of genes encoding resistance to tetracyclines (oral minocycline, Fig. 2b) in the patient’s gut microbiome, highlighting the unintended outcomes of long-term antibiotic exposure. This implicates the host ecosystem as a reservoir of AMR that could present as a major risk factor for subsequent disease(s) and exacerbate AMR transmission. While phages were detected by end-point PCR in patient sera samples to concentrations as high as 166 ng per PCR reaction (Supplementary Table 5), our shotgun metagenomic analyses were unable to detect whole-genome nucleic acid material associated with the AB-SA01 phage genomes across patient microbiome samples. Serial *S. aureus* isolates were tested for phage sensitivity and there was no detectable resistance development (Supplementary Table 1); this further supports the viability of adjunct BT as a non-antibiotic alternative to treating multidrug-resistant bacterial infections. Our microbiome analyses highlight several key considerations when implementing a multi-omics approach to understanding phage therapy in clinical settings. For example, the depth of metagenomic sequencing required to quantitatively track temporal changes in phage abundance is cost prohibitive for routine analysis and needs to be supplemented with quantitative PCR assays (in place of end-point PCR) targeting the conserved regions of the primase genes of the three AB-SA01 phages [12]. Future studies need to include whole-genome sequencing (WGS) of bacterial isolates; for example, WGS data from patient’s methicillin sensitive *S. aureus* isolate collected over a time-series could facilitate comparative genomics to track the succession of variant acquisition (e.g., single nucleotide polymorphisms) in response to BT. This has implications towards understanding the molecular mechanisms driving resistance to phage activity and critically informs phage-cocktail designs.

## Conclusions

Although we assessed only one patient in this study, the safety and efficacy of AB-SA01 was shown to have no adverse reactions (measured by host inflammatory responses and clinical outcome) when treating 13 patients in an Australian hospital with severe *S. aureus* infections [12], as well as in a pilot trial for chronic rhinosinusitis [13]. Our report presents a proof-of-principle framework demonstrating that clinical use of *S. aureus* BT may have minimal collateral damage to the patient’s microbiome—especially the gut microbiome. This may be an important benefit vis a vis systemic antibiotics for treatment of infections, as alterations in gut flora can be associated with multiple adverse events including *Clostridiodes difficile* colitis and increase in multidrug resistant organisms. The effect of BT on the microbiome will need to be assessed in prospective phage therapy trials and warrants investigation specifically as a microbiome sparing therapeutic approach.

## Methods

Samples for microbiome analyses, including samples representative of gut, saliva, and skin (nares, axillary, and forehead) microbiomes, were stored at −20°C and brought to weekly research clinic visits by the patient. The swabs were delivered to the research team and stored at − 20°C until processed for high throughput sequencing and metabolomic analyses. Samples were processed for amplicon 16S rRNA gene sequencing following protocols from the Earth Microbiome Project using primers targeting the V4 hypervariable region (515F barcoded 5′-AATGATACGGCGACCACCGAGATCTACACGCT XXXXXXXXXXXX TATGGTAATT GT GTGYCAGCMGCCGCGGTAA-3′; and 806R 5′-CAAGCAGAAGACGGCATACGAGAT AGTCAGCCAG CC GGACTACNVGGGTWTCTAAT-3′) [14], and metabolite profiling following protocols detailed in the supplementary material compatible with understanding the microbiome response(s) to phage therapy. Unsupervised longitudinal analyses including pairwise distances and LME statistical testing were computed using q2-longitudinal [15]. The LME models with log-ratio as the response variable were carried out with treatment period (i.e., baseline/treatment), time in days, and current treatment as the predictor variables. The LME models with distance from baseline as the response variable were the same but without the treatment period (i.e., baseline/treatment) as predictor variable. Fecal samples were additionally processed for shotgun metagenomics and sequenced on a MiSeq system (Illumina Inc., San Diego, CA, USA) (300 cycles) following manufacturer’s protocol. Amplicon sequencing data were processed using the Qiita [16] platform and QIIME2 [17] software, while metabolomic data were processed using the GNPS platform [18]. Metabolomics methodologies are detailed extensively in the supplementary file. All amplicon sequence data were filtered for blooms [PMID: 28289733] for cross-study assessments. The presence of acquired AMR genes was identified in silico using ABRicate [19] on shotgun metagenomic contig data.

Phage therapy was administered under a single use IND obtained from the FDA and under local regulatory authorization of UCSD Human Research Protection Program (IRB). The patient signed informed consent for phage therapy and for research sample collection, including microbiome analyses. PCR assays for bacteriophages were conducted by Armata Pharmaceuticals (formerly AmpliPhi Biosciences) using proprietary and confidential methods. Patient microbiome and metabolomic data for this current study are deposited to publicly available databases: microbiome study number 10317 (qiita.ucsd.edu) and metabolomics, MSV000083300 (massive.ucsd.edu). The published AB-SA01 phage genomes are publicly available [20].

## Supplementary Information

**Additional file 1: Supplementary figure 1. Supplementary figure 2. Supplementary figure 3. Supplementary figure 4. Supplementary Table 1. Supplementary Table 2. Supplementary table 5.** METHODS—Metabolomics.

**Additional file 2: Supplementary table 3.** Annotation information from GNPS analysis of metabolomic data.

**Additional file 3: Supplementary table 4.** Annotation information from GNPS analysis of metabolomic data, including weblinks to information regarding molecular network components.

## Data Availability

Patient microbiome and metabolomic data for this current study are deposited to publicly available databases: microbiome study number 10317 (qiita.ucsd.edu) and metabolomics, MSV000083300 (massive.ucsd.edu).

## Abbreviations

AGP: American Gut Project
AMR: Antimicrobial resistance
GNPS: Global Natural Products Social Molecular Networking
ICU: Intensive care unit
IV: Intravenous
LME: Linear mixed effects
PCR: Polymerase chain reaction
QIIME: Quantitative Insights Into Microbial Ecology
rRNA: Ribosomal ribonucleic acid
sOTU: Sub-operational taxonomic unit
WGS: Whole-genome sequencing
