# Supplementary material for: Assessment of the microbiome during bacteriophage therapy in combination with systemic antibiotics to treat a case of staphylococcal device infection
Source: Microbiome. 2021 Apr 14;9:92. doi: 10.1186/s40168-021-01026-9 (PMC8048313; doi:10.1186/s40168-021-01026-9)
Supplement: Supplementary file 2 — Additional file 1: Supplementary figure 1. Supplementary figure 2. Supplementary figure 3. Supplementary figure 4. Supplementary Table 1. Supplementary Table 2. Supplementary table 5. METHODS—Metabolomics. [file 40168_2021_1026_MOESM2_ESM.docx]

**Title:** Assessment of the microbiome during bacteriophage therapy in combination with systemic antibiotics to treat a case of staphylococcal device infection

**Authors:** Andre Mu^1,2,^, Daniel McDonald^3^, Alan K. Jarmusch^4,5^, Cameron Martino^3,6,7^, Caitriona Brennan^3^, MacKenzie Bryant^3^, Gregory C. Humphrey^3^, Julia Toronczak^3^, Tara Schwartz^3^, Dominic Nguyen^3^, Gail Ackermann^3^, Anthony D’Onofrio,^8^ Steffanie A. Strathdee^9^, Robert T. Schooley^9^, Pieter C. Dorrestein^3,4,5,7^, Rob Knight^3,7,10,11,#^, Saima Aslam^9^

^1^Doherty Applied Microbial Genomics, Department of Microbiology and Immunology at the Peter Doherty Institute for Infection and Immunity, University of Melbourne, Australia

^2^Microbiological Diagnostic Unit Public Health Laboratory, Department of Microbiology and Immunology at the Peter Doherty Institute for Infection and Immunity, University of Melbourne, Australia

^3^Department of Pediatrics, University of California San Diego, United States

^4^Skaggs School of Pharmacy and Pharmaceutical Sciences, University of California, United States

^5^Collaborative Mass Spectrometry Innovation Center, Skaggs School of Pharmacy and Pharmaceutical Sciences, University of California San Diego, United States

^6^Bioinformatics and Systems Biology Program, University of San Diego, United States

^7^ Center for Microbiome Innovation, University of California San Diego, United States

^8^Antimicrobial Discovery Center, Department of Biology, Northeastern University, United States

^9^Division of Infectious Diseases and Global Public Health, Department of Medicine, University of California San Diego, United States

^10^Department of Bioengineering, University of California San Diego, United States

^11^Department of Computer Sciences and Engineering, University of California San Diego, United States


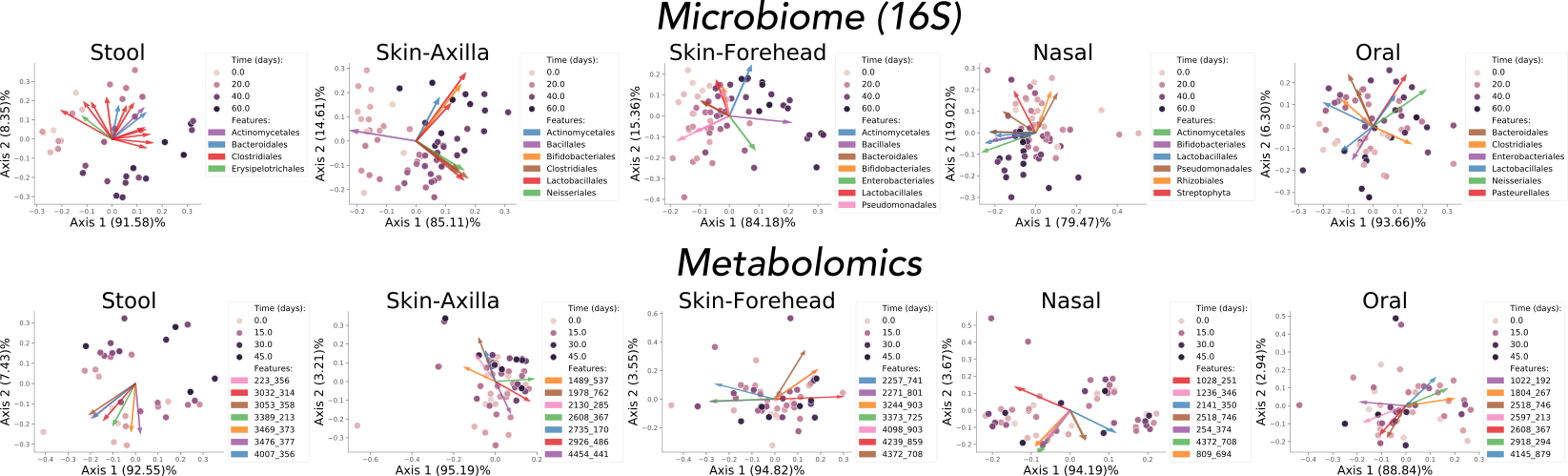


**Supplementary figure 1.** Biplots (2D) for 16S and metabolomic data across each body site. Samples (represented by dots) are colored by sample point, while features (represented by arrows) are colored by taxonomy (16S rRNA gene) or chemical class (metabolite).


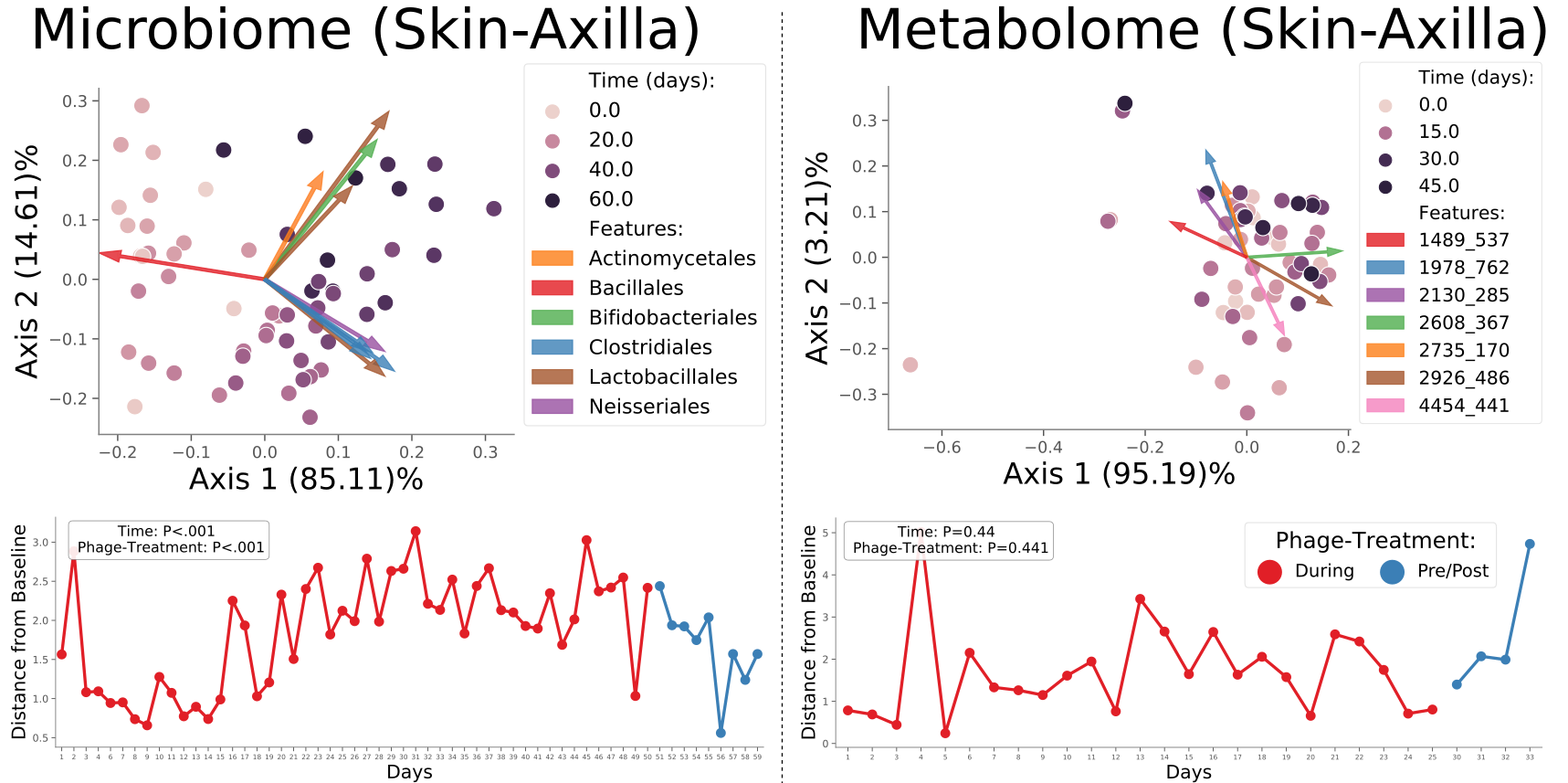


**Supplementary figure 2**. Analysis of the skin-axilla samples. **Microbiome**: The microbiome demonstrated significant temporal trends (P<0.001) and phage-treatment responses (P<0.001) in the first distances. The log-ratio of the exact sequence variant from the red arrow in the biplot of the genus *Staphylococcus* (order *Bacillales*) and a reference of the highly proliferative microbe (orange arrow in the biplot) of the genus *Corynebacterium* (order *Actinobacteria*) showed significant changes in time (P<0.001) and phage-treatment (P<0.001; *c.f.,* Figure 2 of main text).

*Numerator sequence:*

TACGTAGGTGGCAAGCGTTATCCGGAATTATTGGGCGTAAAGCGCGCGTAGGCGGTTTTTTAAGTCTGATGTGAAAGCCCACGGCTCAACCGTGGAGGGTCATTGGAAACTGGAAAACTTGAGTGCAGAAGAGGAAAGTGGAATTCCATG

*Denominator sequence:*

TACGTAGGGTGCGAGCGTTGTCCGGAATTACTGGGCGTAAAGGGCTCGTAGGTGGTTTGTCGCGTCGTCTGTGAAATTCCGGGGCTTAACTCCGGGCGTGCAGGCGATACGGGCATAACTTGAGTACTGTAGGGGTAACTGGAATTCCTG

**Metabolome**: A weak and insignificant (P=0.44) trend in global metabolome profile over time and phage-treatment. However, significant temporal and phage-treatment responses (P=0.03) are observed by selecting for metabolites with opposing loadings in the biplot that vary in relation to this weak time component and calculating log-ratios (*c.f.,* Figure 2 of main text).

Briefly, the beta-diversity analysis was computed using DEICODE

(<https://msystems.asm.org/content/4/1/e00016-19>). The longitudinal analysis, including pairwise distances and LME statistical tests were computed through q2-longitudinal (<https://msystems.asm.org/content/3/6/e00219-18>). All plotting and visualisations were generated using Emperor (<https://www.ncbi.nlm.nih.gov/pmc/articles/PMC4076506/>) and matplotlib.

**Supplementary figure 3.** (A) Log-ratio of sOTU Staphylococcus, and reference skin commensal, Corynebacterium across the pre-, during-, and post-phage sampling points (Sampling point in days). Further, the values given in parentheses indicate the level of confidence in taxonomic classification. (B) Log-ratio of metabolites with opposing loads across pre-, during-, and post-phage sampling points (Sampling point in days).

**Supplementary figure 4**. **Multi-omic plot** Longitudinal rolling mean (window size of 6) of log-ratios of metabolite Class 1 and Class 2, and skin microbes *Staphylococcus* and *Corynebacterium*. The numbers in parentheses indicate the confidence in taxonomic classification for that given exact variant OTU.

**Supplementary Table 1.** Patient *Staphylococcus aureus* isolates and bacteriophage susceptibility testing as previously published.

| **Week of phage Rx** | **Sternal culture** | **AB-SA01 susceptibility^#^** |
| --- | --- | --- |
| **-3 weeks** | *Staphylococcus aureus* | Yes (to all 3 phages) |
| **-2 days** | *S. aureus* | - |
| **Week 1** | Negative | - |
| **Week 2** | Negative | - |
| **Week 3** | *S. aureus, S. epidermidis* | Yes (to all 3 phages) |
| **Week 4 (EOT)** | Negative | - |
| **Heart Transplant**  **(1 week later)** | 2/8 cultures – *S. aureus* | Yes (to all 3 phages) |

^#^Isolates were sent to Armata Pharmaceuticals (formerly AmpliPhi Biosciences, and manufacturer of the phage product, AB-SA01) for phage susceptibility testing as reported in the original case report, Aslam et al 2019

**Supplementary Table 2.** P-values for pairwise Kruskal-Wallis comparisons of Shannon’s Index values across the different phage patient sample types. Number of samples per group given in parentheses.

| **Group 1** | **Group 2** | **P-value** |
| --- | --- | --- |
| **Forehead (n = 45)** | Mouth (n = 60) | 0.015 |
|  | Nares (n = 16) | 1.6E-5 |
|  | Skin (n = 51) | 2.5E-5 |
|  | Stool (n = 31) | 1.02E-10 |
| **Mouth** | Nares | 3.7E-5 |
|  | Skin | 5.6E-4 |
|  | Stool | 1.10E-13 |
| **Nares** | Skin | 0.0030 |
|  | Stool | 0.26 |
| **Skin** | Stool | 1.7E-5 |

*Please refer to comma separated value file for Supplementary table 3*

**Supplementary table 3**. Annotation information from GNPS analysis of metabolomic data.

*Please refer to comma separated value file for Supplementary table 4*

**Supplementary table 4**. Annotation information from GNPS analysis of metabolomic data, including weblinks to information regarding molecular network components.

**Supplementary table 5.** End-point PCR measurements of bacteriophage DNA concentrations in patient serum samples before, and throughout, AB-SA01 adjunctive therapy. Patient serum samples collected pre-phage treatment and at 15, 30, and 60 minutes post-phage treatment on days 1, 8, 15, 22, and 29.

| **AB-SA01 Phage component** | | | **J-Sa36** | **Sa83** | **Sa8** |
| --- | --- | --- | --- | --- | --- |
| **Day 1** | **Pre-treatment concentrations#** | | Nd* | nd | nd |
|  | **Post-dose concentrations** | **15** | 4.9 | 7.2 | 4.9 |
|  |  | **30** | 3.1 | nd | nd |
|  |  | **60** | nd | nd | nd |
| **Day 8** | **Pre-treatment concentrations** | | 2.9 | 3.2 | Nd |
|  | **Post-dose concentrations** | **15** | 7.6 | 17.1 | 3.1 |
|  |  | **30** | 5.8 | 5.8 | Nd |
|  |  | **60** | 1.7 | nd | Nd |
| **Day 15** | **Pre-treatment concentrations** | | 12.1 | 40.6 | 4.5 |
|  | **Post-dose concentrations** | **15** | 6.3 | 25.1 | 4.5 |
|  |  | **30** | 3.0 | 4.8 | 2.7 |
|  |  | **60** | nd | 2.9 | Nd |
| **Day 22** | **Pre-treatment concentrations** | | nd | 3.6 | Nd |
|  | **Post-dose concentrations** | **15** | 2.6 | 8.3 | 2.9 |
|  |  | **30** | 1.7 | 4.7 | Nd |
|  |  | **60** | 8.2 | 29.3 | 7.0 |
| **Day 29** | **Pre-treatment concentrations** | | 71.2 | 166.0 | 10.8 |

^#^ Concentrations measured by standard densitometry analysis and expressed in nanograms per PCR reaction.

*nd – not detected

**SI METHODS – Metabolomics**

**Materials**

Water (Optima LC-MS grade, W64), acetonitrile (Optima LC-MS grade, A9554), Methanol (HPLC grade, A4524), ethanol (200 proof, E7023), and formic acid (Optima LC-MS grade, A11750) were purchased from Fisher Scientific (Houston, TX, USA). The analytical column (Kinetex C18 1.7 µm, 100 Å, 2.1 mm internal diameter by 50 mm in length) and guard cartridge (SecurityGuard ULTRA Cartridge, UHPLC C18 for 2.1 mm internal diameter columns) were purchased from Phenomenex (Torrance, CA, USA). 96-well plates, Eppendorf® Microplate 96/U-PP (Millipore Sigma, Burlington, MA, USA); 96-well storage mats, Storage Mat IIITM 3080 (Corning, Salt Lake City, UT, USA); 96-deep well plates, Nunc™ 96-Well Polypropylene DeepWell™ Storage Plates (Thermo Fisher Scientific, Waltham, MA, USA); 96-deep well plate mats, Nunc™ 96 Well Caps for 1.0mL Polystyrene DeepWell™ Plates (Thermo Fisher Scientific, Waltham, MA, USA); and sealing film, Zone-FreeTM Sealing Films (ZAF-PE-50), non-sterile (Excel Scientific, Victorville, CA, USA) were used.

**Sample Preparation**

The swab tip of the BD Falcon™ SWUBE™ Collection and Transport System swabs were cut into Nunc™ 96-Well Polypropylene DeepWell™ Storage Plates. Sample barcodes were scanned using a barcode scanner and saved into a Google Sheets spreadsheet generating a record of which sample was positioned in each well of the plate. Skin samples (forehead and axilla) and nasal samples were extracted using ethanol-water (1:1) spiked with 1 µM sulfadimethoxine. Oral samples were extracted using methanol-water (4:1) spiked with 1 µM sulfadimethoxine. And fecal samples were extracted using ethanol-water (9:1) spiked with 1 µM sulfadimethoxine. Samples were placed in different plates according to sample type. 500 µL of solvent, composition based on sample type, was added to each well using a multichannel pipette. The deep well plate was covered with a storage mat and floated in an ultrasonic bath for 5 min. The samples were placed in a 4°C fridge overnight to extract (16 h 45 min). Subsequently, the swabs were removed from each well using tweezers, rinsing in between with ethanol-water (7:3) followed by nanopure water. The swab tips were placed in new deep well plates, capped, and retained for further analysis at -80°C. Extracts were evaporated until dry using a CentriVap Benchtop Vacuum Concentrator (Labconco, Kansas City, MO, USA). The 96-well plate containing the dried extract were covered (96-deep well plate mats, Nunc™ 96 Well Caps for 1.0mL Polystyrene DeepWell™ Plates) and stored at -80°C prior to analysis. Immediately prior to analysis, the dried extract material was resuspended in 250 µL of MeOH-water (1:1) spiked with 2 µM sulfamethizole, sonicated for 5 min, and centrifuged for 5 min at 500 g. 200 µL of extract from each well was transferred into a 96-well plate (Eppendorf® Microplate 96/U-PP) and covered with a plate sealing film (Zone-FreeTM Sealing Films). A swab blank was extracted in methanol-water (1:1) and diluted 10-fold using methanol-water (1:1) prior to analysis.

**Data Acquisition**

Samples were analyzed using an ultra-high performance liquid chromatograph (Vanquish, Thermo) coupled with an Orbitrap mass spectrometer (QExactive, Thermo). Chromatographic separation was carried out on the analytical C18 column with corresponding C18 guard cartridge maintained at 40°C during separation. 10.0 µL of extract was injected per sample. Mobile phase composition was as follows: A, water with 0.1% formic acid (*v/v*) and B, acetonitrile with 0.1% formic acid (*v/v*). Gradient elution was performed as follows: 0.0 min, 5.0% B; 1.0 min, 5.0% B; 7.0 min, 100.0% B; 9.5 min, 100.0% B; 9.6 min, 5% B; 11.0 min, 5%B. Flow rate of 0.5 mL min^-1^ was held constant. Heated electrospray ionization (HESI) was performed in the positive ion mode using the following source parameters: spray voltage, 3500 V; capillary temperature, 380 °C, sheath gas, 60.00 (a.u.); auxiliary gas, 20.00 (a.u.); sweep gas, 3.00 (a.u); probe temperature, 300 °C; and S-lens RF level, 60. Heated electrospray ionization (HESI) was performed in the negative ion mode using the following source parameters: spray voltage, 4000 V; capillary temperature, 380 °C, sheath gas, 52.50 (a.u.); auxiliary gas, 13.75 (a.u.); sweep gas, 2.75 (a.u); probe temperature, 300 °C; and S-lens RF level, 60. Positive and negative mode data (negative mode data not discussed) were collected using the same data-dependent acquisition parameters. MS^1^ scans were collected at 30,000 resolution from *m/z* 150 to 1500 was performed (~7 Hz) with a maximum injection time of 100 ms, 1 microscan, and an automatic gain control target of 1x10^6^. The top 3 most abundant precursor ions in the MS^1^ scan were selected for fragmentation with an *m/z* isolation width of 1.5 and subsequently fragmented with stepped normalized collision energy of 20, 30, and 40. The MS^2^ data was collected at 17,500 resolution with a maximum injection time of 100 ms, 1 microscan, and an automatic gain control target of 5x10^5^. The aforementioned details do not fully describe all settings of the method; therefore, we have provided a copy of the method files in MassIVE (MSV000083300).

**Data Processing**

The QExactive mass spectrometry files (.raw) were converted into .mzXML files using MSConvert (http://proteowizard.sourceforge.net/index.shtml). MZmine2 was used to perform feature finding yielding a data matrix of MS1 features (i.e. m/z and retention time) and associated peak area. Feature-based molecular networking outputs were generated from MZmine2 using the “export to GNPS” module which generates a “quant.csv” which contains the MS1 feature information and a corresponding. mgf file which contains MS2 information linked to the MS1 features when an MS2 was detected. Molecular networking was performed using GNPS (gnps.ucsd.edu). The positive mode data job can be accessed via the following link (<https://gnps.ucsd.edu/ProteoSAFe/status.jsp?task=21aa3e7dc4384db59b5d85d0f9489de8>) and the negative mode data job can be accessed via the following link (<https://gnps.ucsd.edu/ProteoSAFe/status.jsp?task=aa1ce4792e384fc0a05a9819072f2236>). A summary of the methods details is available at the provided GNPS links under the “[Networking Parameters and Written Description](https://gnps.ucsd.edu/ProteoSAFe/result.jsp?task=21aa3e7dc4384db59b5d85d0f9489de8&view=written_description)” option.
